# Association of Functional Polymorphism in *TPH2* Gene with Alcohol Dependence and Personality Traits: Study in Cloninger’s Type I and Type II Alcohol-Dependent Inpatients

**DOI:** 10.3390/genes14020413

**Published:** 2023-02-04

**Authors:** Marcela Konjevod, Mirta Rešetar, Ana Matošić, Lipa Čičin-Šain, Jasminka Štefulj

**Affiliations:** 1 Division of Molecular Biology, Ruđer Bošković Institute, HR-10000 Zagreb, Croatia; 2 Division of Pharmacognosy, University of Vienna, AT-1090 Vienna, Austria; 3 Clinical Department of Psychiatry, Sestre Milosrdnice University Hospital Center, HR-10000 Zagreb, Croatia; 4 School of Dental Medicine, University of Zagreb, HR-10000 Zagreb, Croatia; 5 Department of Psychology, Catholic University of Croatia, HR-10000 Zagreb, Croatia

**Keywords:** alcoholism, addiction, personality, 5-HT, tryptophan hydroxylase 2, SNP

## Abstract

Alcohol dependence (AD) is a complex disorder with a poorly understood etiology. In this study, we investigated the relationship between genetic variation in the *TPH2* gene, which encodes the enzyme responsible for serotonin synthesis in the brain, and both AD and personality traits, with attention to Cloninger’s types of AD. The study included 373 healthy control subjects, 206 inpatients with type I AD, and 110 inpatients with type II AD. All subjects were genotyped for the functional polymorphism *rs4290270* in the *TPH2* gene, and AD patients completed the Tridimensional Personality Questionnaire (TPQ). The AA genotype and the A allele of the *rs4290270* polymorphism were more frequent in both patient groups compared with the control group. In addition, a negative association was found between the number of A alleles and TPQ scores for harm avoidance in patients with type II, but not type I, AD. These results support the involvement of genetic variations of the serotonergic system in the pathogenesis of AD, especially type II AD. They also suggest that in a subset of patients, genetic variation of *TPH2* could potentially influence the development of AD by affecting the personality trait of harm avoidance.

## 1. Introduction

Alcohol dependence (AD), also known as alcohol use disorder or alcohol addiction, is a severe psychiatric condition characterized by excessive and uncontrolled alcohol consumption that leads to devastating consequences, including social and economic difficulties and mental and somatic disorders. It is a multifactorial brain disorder influenced by the interaction of genetic and environmental factors [1]. AD is often comorbid with other psychiatric disorders, such as anxiety, depression, and bipolar disorder, as well as with somatic diseases such as cirrhosis and alcoholic hepatitis [2]. The prevalence of AD in the adult population is about 4%, and it is one of the leading causes of death worldwide [3]. Due to addictive behavior, cognitive impairments, and various somatic and psychiatric comorbidities, AD has an immense impact on the quality of life of those affected and their families, causing major economic, health, and social burdens. 

The pathophysiology of AD involves disturbances in several neurotransmitter systems, including dopamine, serotonin, glutamate, opioid peptides, and γ-aminobutyric acid [4,5,6]. Therefore, available pharmacological treatments (e.g., naltrexone, nalmefene, acamprosate) target the aforementioned neurotransmitter systems. 

There is significant variability among individuals with AD in terms of phenotype and underlying causes of the disorder. Several typologies have been developed to distinguish between different types of AD [7,8,9,10], including that proposed by Cloninger and colleagues (1981) [8,11]. This classification system divides AD into type I and type II based on several factors, including etiopathogenic factors, age of onset, sex distribution, personality traits, and alcohol-related problems. Thus, type I AD is more influenced by environmental factors, tends to develop later in life (after age 25), and affects women and men equally, whereas type II AD has a strong genetic component, develops earlier (before age 25), and affects only men [8]. Characteristic personality traits of type I AD patients include low novelty seeking and high harm avoidance, while type II AD patients display high novelty seeking and low harm avoidance and are prone to antisocial behavior (fighting and violence). Therefore, it is believed that the underlying motivation for alcohol abuse in type I AD patients is mainly to relieve anxiety, while type II AD patients drink primarily to induce euphoria. Patients with type I AD may experience prolonged periods of uncontrolled drinking but may also be able to remain abstinent occasionally. On the other hand, type II AD patients are typically unable to refrain from alcohol consumption [8,11].

There are also differences in the neurobiology of type I and type II AD. For example, type I AD patients are thought to have neurobiological deficits primarily related to dopaminergic transmission, while type II AD patients are thought to have deficits primarily related to serotonergic transmission [5,8]. Type II AD patients have decreased serotonin transporter levels in the brain [12,13] and are thought to have decreased serotoninergic activity [8,14]. In contrast, individuals with type I AD characteristics are thought to have increased dopaminergic activity [8], which aligns with their personality traits. 

The brain serotonin (5-HT) system plays an important role in fine-tuning various behavioral processes, including mood, perception, memory, anger and aggression, stress response, appetite, sleep/circadian cycles, and addiction. Studies have documented the involvement of the serotonergic system in the development of several substance abuse disorders, including AD [15,16,17,18,19,20,21]. Decreased serotonergic transmission and serotonin levels are thought to be associated with excessive alcohol consumption, development of alcohol addiction, and certain types of alcohol-related behaviors such as aggression and impulsivity [19,20,21,22]. Serotonergic transmission is regulated by the orchestrated activity of 5-HT receptors, serotonin transporter (5-HTT), and enzymes involved in serotonin synthesis (tryptophan hydroxylase (TPH)) and degradation (monoamine oxidase (MAO)). Polymorphisms in genes encoding the aforementioned proteins have been investigated in several genetic association studies of AD [16,17,21,23,24,25,26]. Such studies could reveal the molecular basis involved in alcoholism and alcohol-related behaviors, leading to a better understanding and an improved therapeutic approach. For example, a short allele of the *5-HTT* gene has been shown to be associated with AD [25]. Furthermore, associations of polymorphisms in the serotonin receptor 2A gene [24] and in the *TPH1* gene [27] with AD have been reported. However, several studies reported no associations between serotonin-related gene polymorphisms and AD [26,28,29]. Therefore, due to the conflicting results, further studies on the involvement of the serotonergic system in AD are needed to clarify the still unclear etiology of this complex and heterogeneous disorder.

Considering the differences in genetic background and the involvement of the serotonergic system in the pathogenesis of Cloninger’s type I and type II AD, we hypothesized that these two types of AD might have a different association with polymorphisms in serotonin-related genes. Therefore, the aim of this study was to examine the relationship between the above types of AD and the functional polymorphism *rs4290270* in the gene encoding tryptophan hydroxylase 2 (TPH2), the enzyme responsible for serotonin synthesis in the brain. We also aimed to investigate the possible association between the *rs4290270* polymorphism and certain personality traits (novelty seeking, harm avoidance, and reward dependence) in patients with type I and type II AD.

## 2. Materials and Methods

### 2.1. Participants

The study included 689 male participants of Croatian origin, of whom 373 were control subjects and 316 were alcohol-dependent subjects. The control subjects were healthy blood donors with no personal or family history of AD or other psychiatric disorders. The alcohol-dependent subjects were inpatients at Sestre Milosrdnice University Hospital Center, Zagreb, Croatia. The AD was diagnosed by the structured clinical interview based on DSM-V criteria. In addition, alcohol-dependent patients were classified into type I and type II AD according to Cloninger’s typology [8,11]; the number of subjects (n) in each group was 206 and 110, respectively. Classification was performed by an experienced psychiatrist using an assessment protocol that took into account age at AD onset and at first hospitalization for AD, family history of AD, severity of alcohol-related problems, personality characteristics, and coexisting psychopathologies. The Tridimensional Personality Questionnaire (TPQ) was used to measure the personality traits of novelty seeking (NS), harm avoidance (HA), and reward dependence (RD) in AD patients [30]. The TPQ questionnaire is based on the theory that each of the three personality traits is closely related to the activity of a specific monoaminergic neurotransmitter system: NS to dopaminergic, HA to serotonergic, and RD to the noradrenergic system [31,32]. The Short Alcohol Dependence Data (SADD) questionnaire was used to assess the severity of alcohol dependence, with a score of 1–9 being classified as low dependence, 10–19 as moderate dependence, and 20–45 as high dependence [33]. All participants signed an informed consent to participate in the study.

### 2.2. DNA Extraction and Genotyping

DNA was isolated from peripheral blood cells using standard procedures. The genotypes of *rs4290270* polymorphism, located in exon 9 of the *TPH2* gene (chr12:72022455 in GRCh38.p13), were determined using the TaqMan SNP Genotyping Assay and the 7300 Real-Time PCR System (both from Applied Biosystems, Foster City, CA, USA). The assay ID was C_26385365_10, and the PCR solution contained 25 ng of DNA, 5 µL of 2X TaqMan Master Mix, and 0.25 µL of 40X NP genotyping assay in a total volume of 10 µL. Thermal conditions for PCR were as follows: polymerase activation (95 °C, 10 min), followed by 40 cycles of denaturation (92 °C, 15 s) and primer annealing and extension (60 °C, 1 min).

### 2.3. Statistical Analysis

Categorical variables were expressed as number (N) and percentage (%). Continuous variables were expressed as mean and standard deviation (SD) for normally distributed data or as median and interquartile range for non-normally distributed data. Normality of data was assessed using the Kolmogorov–Smirnov test. Continuous variables were compared using the Mann–Whitney U test, the Kruskal–Wallis test, one-way analysis of variance (ANOVA), and two-way ANOVA (with Sidak’s post hoc test), as appropriate. The Hardy–Weinberg equilibrium [34] and differences in genotype distribution between the control and case groups were examined using the chi-square (χ^2^) test. Differences in allele frequencies were determined using Fisher’s exact test (FET). Odds ratios (OR) with 95% confidence intervals (95% CIs) were calculated to assess effect size. The level of statistical significance was set at *p* < 0.05. All statistical analyses were performed using GraphPad Prism version 8 (GraphPad Software, San Diego, CA, USA).

## 3. Results

### 3.1. Characteristics of the Study Participants

The age (mean ± SD) of the 373 healthy control subjects was 44.2 ± 15.2 years. The demographic and clinical characteristics of the type I and type II AD patients are shown in Table 1. As expected, compared with the type I AD patients, the type II AD patients had an earlier onset of alcohol abuse, a higher prevalence of AD in the family history, higher scores for the personality trait of NS, and lower scores for the personality trait of HA (Table 1). In addition, type I and type II AD patients differed in age, education level, marital status, smoking behavior, alcohol dependence level, and frequency of psychiatric and somatic comorbidities (see Table 1 for details).

### 3.2. Association of rs4290270 Polymorphism with Type I and Type II AD

**Figure 1:**
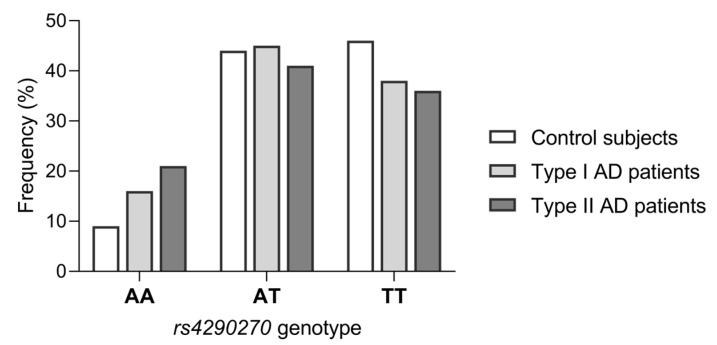
The distribution of *rs4290270* genotypes in 377 healthy control subjects, 206 subjects with type I alcohol dependence (AD) and 110 subjects with type II AD.

The distribution of *rs4290270* genotypes in the control group, type I AD patients, and type II AD patients is shown in Figure 1. In all three study groups, frequencies of *rs4290270* genotypes were in Hardy–Weinberg equilibrium (all *p*-values > 0.05). 

The frequencies of *rs4290270* genotypes were statistically significantly different between type II AD patients and control subjects (*p* = 0.003; χ^2^ = 11.45). However, the comparison between type I AD patients and control subjects showed only borderline statistical significance (*p* = 0.057; χ^2^ = 5.74). Nevertheless, carriers of the homozygous minor allele A genotype were statistically significantly overrepresented in both type I and type II AD patients compared to control subjects (Table 2), although the effect size was larger for type II AD patients (OR = 2.53; CI = 1.42–4.47) than type I AD patients (OR = 1.73; CI = 1.04–2.85). In addition, the frequency of minor allele A was significantly higher in both the type I (OR = 1.34; CI = 1.05–1.73) and type II (OR = 1.59; CI = 1.16–2.16) AD groups compared to the control group (Table 2). There were no statistically significant differences in frequencies of AT and TT genotypes between control subjects and type I or type II AD patients (Table 2). 

No significant differences in the distribution of *rs4290270* genotypes and alleles were found between AD patients subdivided based on the family history of AD, degree of alcohol dependence (assessed by SADD), smoking status, suicidal ideation/attempt, mood disorders, personality disorders, or somatic disorders (all *p*-values > 0.05). 

### 3.3. Association of rs4290270 Polymorphism with Personality Traits

**Figure 2:**
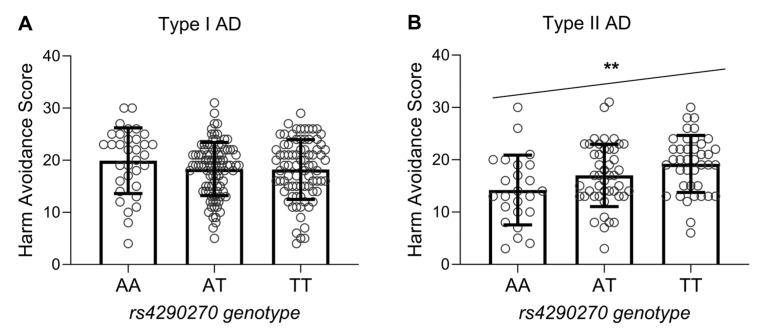
Harm avoidance scores as a function of *rs4290270* genotype in patients with type I (**A**) and type II (**B**) alcohol dependence. Shown are individual values and group means with standard deviations. ** *p =* 0.002 (test for linear trend).

The *rs4290270* polymorphism was not associated with the personality traits of NS and RD in either type I or type II AD patients (Table 3). However, the *rs4290270* polymorphism was found to be associated with the personality trait of HA in type II AD patients. Specifically, type II AD patients homozygous for allele A had the lowest HA scores, and those homozygous for allele T had the highest HA scores, while such a trend was not observed in type I AD patients (Figure 2). To further corroborate these findings, we also conducted analyses using the two-factor ANOVA. The results showed a statistically significant interaction between *rs4290270* genotype and type of AD on HA scores (*p* = 0.002, F_2,310_ = 6.29), with the post hoc test showing that *rs4290270* affected HA scores only in patients with type II AD (*p* = 0.002 for the difference between AA and TT genotype groups), but not in patients with type I AD (*p* > 0.05 for all comparisons). The post hoc analysis also revealed that only carriers of the AA genotype had decreased HA scores in type II compared to type I AD (*p* = 0.0007), while this was not the case for carriers of the AT or TT genotype (*p* = 0.05). For NS scores, only the main effect of type of AD was statistically significant (*p* < 0.0001), while no significant interaction or main effects of genotype or type of AD was found for RD scores (all *p* > 0.05).

## 4. Discussion

In the present study, we found that the frequencies of genotypes and alleles of the *rs4290270* polymorphism in the *TPH2* gene differed between healthy control subjects and alcohol-dependent patients, supporting the idea that variations in the serotonergic system may play a role in the development of AD. Additionally, the observed differences were more pronounced in patients diagnosed with Cloninger’s type II than type I AD, suggesting that genetic variants of *TPH2* may be more influential in determining type II AD. Furthermore, *TPH2* variants were found to be associated with the personality trait of HA in type II AD patients, but not in type I AD patients, supporting the different biological mechanisms underlying the two types of AD.

TPH2 is a rate-limiting enzyme of the serotonin synthesis pathway in the brain and therefore plays a key role in regulating serotonergic transmission [35,36,37]. Previous research has shown that TPH2 protein and *TPH2* mRNA levels are altered in the brains of AD patients, with higher levels found in the dorsal and median raphe nuclei of AD patients than of healthy controls [38]. This upregulation in *TPH2* expression was more pronounced in AD patients with a positive family history of alcohol abuse, suggesting that genetic factors may contribute to the observed alteration [38]. Overall, these findings highlight the possible important role of the *TPH2* gene in the pathogenesis of AD.

The *TPH2* gene, located on chromosome 12 (12q21.1), contains a number of single-nucleotide polymorphisms (SNPs) in its promoter, intron, and coding regions [23,39,40,41,42,43,44,45,46], some of which may potentially affect the *TPH2* regulation and function. The *rs4290270* polymorphism investigated in this study is a synonymous A-to-T substitution in exon 9, which means that it does not alter the amino acid sequence of the TPH2 protein. However, *rs4290270* has been associated with changes in *TPH2* mRNA levels in the human pons [39] and with alternative splicing of *TPH2* transcripts in the human amygdala [41]. This alternative splicing produces two *TPH2* variants that have been identified in the human amygdala, denoted *TPH2a* and *TPH2b*. The *TPH2b* variant, which encodes a protein with higher enzymatic activity, was found to be absent in the presence of the *rs4290270* minor allele A, although the underlying mechanisms behind this effect of *rs4290270* on splicing and enzymatic activity remain unclear [41]. 

In addition, several previous studies have reported the association of the *rs4290270* polymorphism with mental disorders and traits, further supporting its functional role in modulating serotonergic transmission. For example, the AA genotype of *rs4290270* has been associated with suicidal behavior [41] and higher self-reported depression scores [45], while the A allele has been associated with more positive subjective effects of cocaine in cocaine-dependent individuals [44] as well as with depressive and anxiety traits in AD patients [23].

In our study, we found a significantly higher frequency of both AA genotypes and A alleles of the *rs4290270* polymorphism in type I and type II AD inpatients compared to healthy controls without personal or family history of alcohol abuse and other mental illnesses. As mentioned previously, the A allele of *rs4290270* has been associated with the absence of splicing variant with a higher enzymatic activity [41]. Therefore, our findings provide further evidence for a role of decreased serotonergic activity in the development and manifestation of AD in some individuals. It is worth noting that the observed alterations in the distribution of *rs4290270* variants were more pronounced in patients with type II than type I AD, the type of AD believed to be more genetically determined and predominantly associated with deficits in serotonergic transmission [8,14]. 

There are several other studies that have examined the possible association between *TPH2* variants and AD. Zill et al. (2007) investigated 20 intronic SNPs in the *TPH2* gene and reported that neither individual SNPs nor haplotypes were significantly associated with AD [46]. Some possible reasons for the discrepancy with our positive results could be related to the ethnic background (German in [46] vs. Slavic in our study), the severity of alcohol dependence (alcohol-dependent outpatients in [46] vs. inpatients, which could represent a more severe form of AD, in our study), or the variants studied (intronic SNPs in [46] vs. *rs4290270*, a putative functional [39,41] exonic SNP, in our study). 

Furthermore, Plemenitaš et al. (2015) investigated the *rs4290270* polymorphism together with three other *TPH2* polymorphisms (*rs4570625*, *rs1843809*, and *rs7305115*, located in promoter, intron, and exon regions, respectively) in a sample of 101 alcohol-dependent and 97 healthy subjects [23]. In a single-variant analysis, none of the SNPs studied were associated with AD [23]. Two other studies also reported no association between the *TPH2* promoter variant *rs4570625* and AD [16] or alcohol consumption [29]. However, Plemenitaš et al. (2015) found that *TPH2* haplotypes including *rs4290270*, *rs4570625*, and the other two SNPs studied were significantly differentially distributed between alcohol-dependent patients and healthy controls [23]. This suggests an important aspect of the interaction of the *rs4290270* polymorphism with other polymorphisms in the *TPH2* gene in predisposing to the development of AD. The *rs4290270* polymorphism has been reported to interact with genetic variants also in other genes in influencing behavioral disorders—specifically, with polymorphism in the *TPH1* gene (encoding a peripheral isoform of TPH) in influencing heroin addiction [43]. Clearly, further studies involving *rs4290270* and other polymorphisms are needed to elucidate the role of *TPH2* in a complex genetic background of AD.

Personality traits such as NS, HA, and RD are thought to have a significant impact on the development of the different types of AD [5]. These traits, which are relatively stable and shape a person’s behavior and thoughts, are largely influenced by genetics and neurobiology. Previous research has suggested that genetic variations in the serotonin system may influence personality traits. In particular, the *TPH2* promoter polymorphism *rs4570625* has been found to influence HA, but not NS and RD, in healthy individuals [47,48]. However, the relationship between *TPH2* variants and personality traits in individuals with AD has not been previously examined.

Our study is the first to show that genetic variation in the *TPH2* gene has an effect on HA in patients with type II AD. Specifically, the A allele of the *rs4290270* polymorphism, which was associated with lower enzymatic activity [41] and was here overrepresented in AD patients compared to control subjects, was associated with lower HA scores in patients with type II AD. In contrast, the *rs4290270* polymorphism did not affect HA scores in patients with type I AD, who had significantly higher HA scores than patients with type II AD. Moreover, the reducing effect of the AA genotype on HA scores in patients with type II AD appeared to be solely responsible for the lower HA scores in this group compared to patients with type I AD. These results suggest that genetic variants in the serotonin system may play a more influential role in HA in patients with type II AD than in patients with type I AD. They also highlight the importance of considering the influence of genetic variation on personality traits in individuals with different types of AD and suggest that further studies are needed to fully understand the relationship between *TPH2* variants and personality traits in this population.

There are several questions that need to be explored in future research. It is unclear what drives the differences in the sensitivity of HA to genetic influences and whether the presence/absence of this influence is specific to a particular type of AD or varies across other diagnostic categories, including healthy individuals. In addition, it is important to understand how this effect may contribute to the development of AD.

This study has several strengths. It is the first study to investigate *TPH2* variants in two homogeneous samples of male inpatients with type I and type II AD. All participants were of Croatian origin, reducing the potential confounding effect of population stratification. A putative functional SNP was studied.

However, there are also some limitations, including a relatively small sample size for genetic research, particularly in the case of patients with type II AD (n = 110). In addition, other polymorphisms in *TPH2* and other genes should be studied to better understand the role of *TPH2* in the pathophysiology of AD. The genetic background of AD is complex, with multiple genes having a small effect on specific endophenotypes. In addition, gene–gene interactions are important in behavioral phenotypes such as AD as well as temperament traits. In a sample of healthy individuals from the Finnish general population, more than 700 genes have been identified to influence human temperament, explaining almost all of the heritability expected from twin studies [49]. Although *TPH2* was not identified in the corresponding genome-wide association study [49], it may still be relevant for certain ethnic populations or for patients with AD who have a distinct genotypic and environmental background that differs from that of the general population.

Therefore, the role of *TPH2* in the pathogenesis of AD is still unclear, and further research is needed to better understand its contribution. Despite its limitations, this study can be considered preliminary and should encourage validation of the results in a larger study with genome-wide evaluation and a more comprehensive assessment of temperament and character.

## Figures and Tables

**Table 1:** Demographic and clinical characteristics of type I (n = 206) and type II (n = 110) alcohol-dependent patients.

Characteristics		Type I	Type II	*p*-Value
Age (years)		50 (9)	31 (7)	**<0.0001 ^a^**
NS score		14 (7)	18 (6)	**<0.0001 ^a^**
HA score		19 (8)	17.5 (9)	**0.043 ^a^**
RD score		20 (4)	20 (4)	0.695 ^a^
SADD score		22 (3)	21 (4)	**<0.0001 ^a^**
Age at AD onset (years)	≤25	22 (11.1)	86 (81.1)	**<0.0001 ^c^**
	>25	176 (88.9)	20 (18.9)	
Education	Elementary school	31 (15.1)	21 (19.1)	**0.003 ^b^**
	High school	140 (68.0)	85 (77.3)	
	University	35 (17.0)	4 (3.6)	
Marital status	Married	132 (64.1)	46 (41.8)	**<0.0001 ^b^**
	Unmarried	34 (16.5)	55 (50.0)	
	Divorced	30 (14.6)	9 (8.2)	
	Widower	10 (4.9)	0 (0.0)	
Family history of PD	Yes	12 (5.8)	14 (12.7)	**0.051 ^c^**
	No	194 (94.2)	96 (87.3)	
Family history of AD	Yes	135 (65.5)	85 (77.3)	**0.040 ^c^**
	No	71 (34.5)	25 (22.7)	
Smoking	Yes	140 (68.0)	97 (88.2)	**<0.0001 ^c^**
	No	66 (32.0)	13 (11.8)	
Childhood abuse	Yes	94 (45.6)	62 (56.4)	0.077 ^c^
	No	112 (54.4)	48 (43.6)	
Mood disorders	Yes	64 (31.1)	21(19.1)	**0.024 ^c^**
	No	142 (68.9)	89 (80.9)	
Personality disorders	Yes	7 (3.4)	40 (36.4)	**<0.0001 ^c^**
	No	199 (96.6)	70 (63.6)	
Suicidal ideation/attempt	Yes	31 (15.1)	26 (23.6)	0.066 ^c^
	No	175 (85.0)	84 (76.4)	
Liver and GI tract lesions	Yes	94 (45.6)	23 (20.9)	**<0.0001 ^c^**
	No	112 (54.4)	87 (79.1)	

Continuous variables (age, NA, HA, RD, and SDDD scores) are presented as median with interquartile range in parentheses. Categorical variables are reported as number of subjects with percentage in parentheses. Data about age at onset of alcohol dependence were missing for 8 type I AD patients and 4 type II AD patients. *p*-values were determined by ^a^ Mann–Whitney U test, ^b^ chi-square test, or ^c^ Fisher’s exact test. Statistically significant *p*-values are in bold. NS, novelty seeking; HA, harm avoidance; RD, reward dependence; SADD, Short Alcohol Dependence Data questionnaire; AD, alcohol dependence; PD, psychiatric disorders; n, number of subjects.

**Table 2:** Distribution of genotypes and alleles of the *rs4290270* polymorphism in healthy control subjects and patients with type I and type II alcohol dependence (AD). Numbers of subjects are indicated with percentages in parentheses.

*TPH2 rs4290270*		Type I AD (n = 206)	Type II AD (n = 110)	Controls (n = 373)	Type I AD vs. Controls	Type II AD vs. Controls
Genotypes	AA	33 (16.0)	24 (21.8)	37 (9.9)	0.057 ^a^	**0.003 ^a^**
	AT	93 (45.2)	46 (41.8)	164 (44.0)		
	TT	80 (38.8)	40 (36.4)	172 (46.1)		
Minor allele homozygotes	AA	33 (16.0)	24 (21.8)	37 (9.9)	**0.034 ^b^**	**0.002 ^b^**
	AT/TT	173 (84.0)	86 (78.2)	336 (90.1)		
Heterozygotes	AT	93 (45.2)	46 (41.8)	164 (44.0)	0.794 ^b^	0.743 ^b^
	AA/TT	113 (54.9)	64 (58.2)	209 (56.0)		
Major allele homozygotes	TT	80 (38.8)	40 (36.4)	172 (46.1)	0.097 ^b^	0.083 ^b^
	AT/AA	126 (61.2)	70 (63.6)	201 (53.9)		
Alleles	A	159 (38.6)	94 (42.7)	238 (31.9)	**0.024 ^b^**	**0.004 ^b^**
	T	253 (61.4)	126 (57.3)	508 (68.1)		

*p*-values were determined by ^a^ chi-square test or ^b^ Fisher’s exact test. Statistically significant *p*-values are in bold. n, number of subjects.

**Table 3:** Novelty seeking (NS), harm avoidance (HA), and reward dependence (RD) in patients with type I and type II alcohol dependence (AD) stratified by *rs4290270* genotype. Shown are the medians (with interquartile range in parentheses) of scores obtained with the Tridimensional Personality Questionnaire.

	Type I AD (n = 206)				Type II AD (n = 110)			
Genotype	n	NS	HA	RD	n	NS	HA	RD
AA	33	15 (7)	22 (9)	20 (3)	24	21 (11)	14 (9)	20 (4)
AT	93	14 (7)	19 (7)	20 (5)	46	18 (7)	17 (9)	21 (4)
TT	80	15 (7)	19 (8)	20 (5)	40	19 (6)	20 (8)	19 (5)
*p*-value		0.143 ^a^	0.302 ^b^	0.403 ^a^		0.757 ^a^	**0.006 ^b^**	0.194 ^a^

*p*-values were determined by ^a^ Kruskal–Wallis test or ^b^ one-way ANOVA. Statistically significant *p*-values are in bold. n, number of subjects.

## Data Availability

All data supporting the conclusions of this manuscript are provided in the text and figures.
